# The slow component of oxygen uptake of insulated muscular groups measured with NIRS during intermittent isometric contractions in humans

**DOI:** 10.14814/phy2.70491

**Published:** 2025-07-30

**Authors:** E. Tam, M. Bertucco, C. Capelli

**Affiliations:** ^1^ Section of Movement Sciences, Department of Neuroscience, Biomedicine and Movement Sciences University of Verona Verona Italy; ^2^ Department of Pathophysiology and Transplantation University of Milano Milan Italy

**Keywords:** muscular oxygen uptake slow component, MVC, NIRS, repeated isometric exercise

## Abstract

When exercising in the severe‐intensity domain, oxygen uptake (V̇O_2_) does not reach a steady state since it slowly and continuously drifts due to the appearance of the slow component of V̇O_2_ (V̇O_2sc_). V̇O_2sc_ has been customarily evaluated by measuring V̇O_2_ using pulmonary gas exchanges, including the contribution of the metabolic expenditure of the respiratory muscles, heart, and muscles recruited for stabilizing the posture, etc. We assessed the muscular oxygen uptake (mV̇O_2_, in μM·s^−1^) of flexor digitorum superficialis by using near‐infrared spectroscopy (NIRS) during brief arterial occlusions imposed over repeated, cyclic isometric hand grips performed at two different percentages (25%; 50%) of maximal voluntary contraction (MVC). mV̇O_2_ was significantly larger at 50% MVC than at 25% MVC at all the time points (*p* < 0.05) as expected, apart from the values at the 3rd minute. Secondly, mV̇O_2_ increased linearly at 25% of MVC (mV̇O_2_ = 1.73 + 0.108 × min; *r* ^2^ = 0.993; *p* < 0.0033) and at 50% of MVC (mV̇O_2_ = 2.41 + 0.240 × min; *r* ^2^ = 0.956; *p* < 0.0223) from the 3rd minute of exercise onwards; the slopes of the two linear regressions were significantly different (*p* < 0.0232). We suggest that the V̇O_2sc_ of mV̇O_2_ occurring during intermittent isometric contractions can be assessed with NIRS during brief complete arterial occlusions regularly interspersed in the series of contractions. In addition, the technique can discriminate the rates of increase of mV̇O_2_ corresponding to different percentages of MVC.

## Graphical abstract

The slow component of muscle oxygen consumption can be assessed with NIRS during brief arterial occlusions.

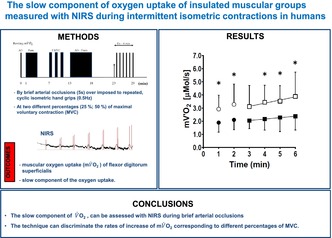

## 1 INTRODUCTION

When exercising in the severe‐intensity domain, oxygen uptake (V̇O_2_) does not remain at a steady state since it slowly and continuously drifts to reach the maximum (V̇O_2max_) due to the appearance of the so‐called slow component of V̇O_2_ (V̇O_2sc_) (Poole & Jones, 2012). The physiological basis of this phenomenon remains a matter of investigation and is subject to debate. Having established beyond any reasonable doubt that V̇O_2sc_ is predominantly of muscular origin (Poole et al., 1991), it has also been suggested that it is likely the effect of the progressive recruitment of the less efficient Type II fibers characterized by a more costly aerobic synthesis of ATP per mole of utilized O_2_. Conversely, recent data obtained on muscles stimulated in situ (Grassi et al., 2015; Zoladz et al., 2008; Zoła̧dz & Korzeniewski, 2001) and in human volunteers (Tam et al., 2024) suggested that intrinsic mechanisms occurring inside the contracting fibers may elicit, or at least substantially contribute to, V̇O_2sc_ due to decreasing muscular efficiency or an increase of O_2_ muscular uptake for the same rate of ATP production.

V̇O_2sc_ has been customarily evaluated in exercising humans by measuring V̇O_2_ using pulmonary gas exchanges, including, the contribution of the respiratory muscles, heart, and muscles recruited for stabilizing posture. This approach may make it difficult, if not even impossible, to evaluate the possible effects on muscular V̇O_2sc_ of interventions designed to manipulate the number of motor units recruited in a motor task performed at a constant intensity using selected and small muscle groups. Muscle deoxygenation kinetics have been extensively studied using NIRS during dynamic and static exercises (Hamaoka et al., 1996; Van Beekvelt et al., 2002; Bringard & Perrey, 2004; Layec et al., 2009; Ryan et al., 2012). During repeated isometric contractions, deoxyhaemoglobin concentration (HHb) progressively increases with contraction intensity due to accumulating metabolic stress and reduced oxygen delivery relative to demand (Van Beekvelt et al., 2002). These studies showed that HHb kinetics reflect local oxygen extraction and utilization, which are influenced by contraction intensity and duration, providing a basis for investigating muscular oxygen uptake (mV̇O_2_) in small muscle groups. This approach was utilized by several groups (Adami et al., 2017; Pilotto et al., 2022; Ryan et al., 2012, 2014; Willingham & McCully, 2017; Zuccarelli et al., 2020, 2021) in determining muscle mV̇O_2_ kinetics in the recovery following exercise. We adopt and adapt the basic methodological principle.

The aim of this study, summarized in the present report, consisted of describing the feasibility of assessing muscular oxygen uptake (mV̇O_2_) of *flexor digitorum superficialis* (FDS) assessed by using NIRS during brief arterial occlusions over imposed to repeated, cyclic isometric hand grips performed at two different percentages of maximal voluntary contraction (MVC). Data will show that assessing and quantitatively describing the time course of mV̇O_2sc_ during repeated contractions was possible.

## 2 METHODS

### 2.1 Subjects

Ten moderately active young men volunteered for the study (24.6 y/o ± 1.6; 1.78 m ± 0.04; 74.6 kg ± 8.7). Each participant signed an informed consent form before entering the study. In the recruitment, we applied inclusion and exclusion criteria from the American College guidelines (2014). The investigation has been conducted following the ethical standards of the Declaration of Helsinki. It has been approved by the author's institutional review board (*Comitato di Approvazione della Ricerca sulla Persona*—C.A.R.P.—n. 19.R2/2021 approval on 26th October 2021).

### 2.2 Experimental design, protocol

The experiments were carried out at the Exercise Physiology Laboratory of the University of Verona, Italy. Each session lasted approximately two and a half hours, held in a single meeting in the laboratory. Before the experiments, we measured the body weight of the volunteers and adipose tissue thickness (ATT) by computing half of the skinfolds of the area explored by the NIRS probe over the surface of the superficial flexor muscle (average of three repeated measurements).

The subject sat before a table, and the upper limb was positioned on a soft support. The NIRS probe was placed on the muscle belly and secured and protected from light interference. The inflating cuff was proximal to the probe.

Resting mV̇O_2_ was then assessed by inflating the cuff (300 mm Hg) for 10 s. Then, after a 1‐min recovery period, the volunteer performed a 3‐min familiarization phase, engaging in intermittent, submaximal contractions using an electronic handgrip dynamometer.

After this phase, the volunteer performed three maximal voluntary contractions (MVCs), each lasting 5 s, with a 1‐min recovery period in between. The highest MVC value was then selected, and 25% and 50% of the MVC were calculated and used as the target strengths for the following series of intermittent contractions. We selected the exercise intensities of 25% and 50% maximal voluntary contraction (MVC), as did previous studies using isometric protocols (Bringard & Perrey, 2004; Grassi et al., 2015; Van Beekvelt et al., 2002).

**FIGURE 1:**
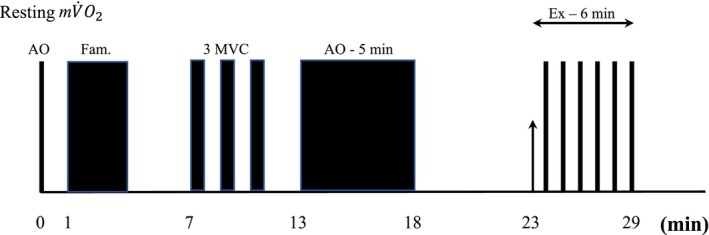
Graphical description of the experimental protocol. AO, arterial occlusions; Fam., familiarization phase; MVC, maximal voluntary isometric contractions. Arterial occlusions (AO) were applied every 50 s during the exercise. Please see the text for further details.

At the end of this phase, we inflated the cuff at 300 mm Hg for 5 min, imposing arterial occlusion to obtain complete deoxygenation of the tissue explored by the probe. After 5 min of recovery, the volunteer performed intermittent, submaximal, isometric voluntary contractions (1 s of contraction, 1 s of recovery, duty cycle 1 s) at 25% of individual MVC. The exercise lasted 6 min, and every 50 s, the volunteer interrupted the exercise, relaxing the muscles while an arterial occlusion (300 mm Hg) of 5 s was applied to measure mV̇O_2_. After 6 min of recovery, the exercise protocol was repeated using an isometric strength corresponding to 50% MVC (Figure 1).

### 2.3 Data acquisition and treatment

Skinfolds were measured using a mechanical caliper (Holtein, UK), and body weight was assessed using a scale (Tanita BWB‐800, Wunder SA, USA).

Handgrip isometric strength was measured using a grip force transducer (MLT004/ST, AD Instruments, Dunedin, NZ) connected to an AD converter‐acquisition system (Power Lab, AD Instruments, Dunedin, NZ) run by a patented software (LabChart, AD Instruments, Dunedin, NZ). The acquisition rate was set to 100 Hz, and the digitized strength signal (N) was displayed on a video screen for the participants and operators.

NIRS data were collected at 10 Hz. The NIRS (Portamon, Artinis Medical System B.V., The Netherlands) signals collected included oxygenated hemoglobin (O_2_Hb), deoxygenated hemoglobin (HHb), and total blood volume (tHb) (tHb = O_2_Hb + HHb). The three transmitters each emitted two wavelengths (760 nm and 850 nm) of light at three separate transmitter‐receiver distances (30 mm, 35 mm, and 40 mm). We performed all data analysis on the 40‐mm transmitter–receiver channel because it provided optimal light penetration depth (~20 mm) to reach the deeper layers of the flexor digitorum superficialis.

Arterial occlusions were imposed by inflating a cuff using E20 plus Compressor AG101 (Hokanson, USA). By inflating the cuff (SC10D) to a pressure of 300 mmHg, we prevent arterial blood perfusion and venous blood efflux in and from the tissue explored by the NIRS probe.

The progressive increase of HHb measured during the ischemic occlusions was used to estimate mV̇O_2_ (Ryan et al., 2012; Van Beekvelt et al., 2001). To this aim, raw HHb data were exported after the experiment to Microsoft Excel (Microsoft, USA) and corrected for blood volume changes as suggested by Ryan and colleagues (Ryan et al., 2012). Afterward, data were analyzed with the software AcqKnowledge (Biopac Systems Inc., USA), which calculated the linear rate of increase of the corrected [HHb] as a function of the time of occlusion (∆HHb/∆t). mV̇O_2_ derived from ∆HHb/∆t was expressed in μM·s^−1^ (μmol/(L·s)) (Binzoni et al., 2000; Lucero et al., 2018; Van Beekvelt et al., 2001). The rate of increase in mV̇O_2_ was quantified by fitting a linear regression to mV̇O_2_ values measured from minute 3 to minute 6 during intermittent isometric contractions at 25% and 50% MVC. The slope of this regression line represents the rate of increase in mV̇O_2_ μM·s^−1^ and reflects the V̇O_2sc_ at the muscle level. The first 2 min were excluded from this analysis to allow for the stabilization of mV̇O_2_ values following the initial transient phase.

### 2.4 Statistics

Data are presented as mean plus/minus S.D. We tested for normality using the D'Agostino‐Pearson omnibus and Shapiro–Wilk tests; the effect size assessment was calculated using Cohen's *d*, pooled. We performed a repeated‐measures two‐way ANOVA by a Mixed effects model to analyze the effects of exercise intensity (25% vs. 50% MVC) and time (six epochs: 1–6 min) on mV̇O_2_. Mauchly's test assessed sphericity, and Greenhouse–Geisser corrections were applied when sphericity was violated. Post hoc pairwise comparisons were conducted using Fisher's LDS test to identify specific differences between conditions and time points. We assessed the slow component of mV̇O_2sc_ by fitting (Motulsky & Christopoulos, 2004) the mean values of *m*V̇O_2_ as a function of time from the third to the sixth minutes of exercise performed at 25% and 50% of MVC using linear regressions calculated by the least‐squares residuals method. The difference between the slopes of the two regressions was assessed as indicated by Zar, 2010. *α* value was set at 0.05; statistical analysis was performed using Prism, version 10.0 for MacIntosh (GraphPad Software, Boston, MA, USA).

## 3 RESULTS

Average ATT over FDS amounted to 5.4 mm ± 2.4. MVC was 39.4 kg ± 12.0, and 50% of MVC and 25% of MVC were 19.7 kg ± 6.0 and 9.9 kg ± 3.0, respectively.

mV̇O_2_ was significantly larger at 50% MVC than at 25% MVC at all the time points (*p* < 0.05), apart from the values at the third minute (0.05 < *p* < 0.1). (Table 1).

**TABLE 1:** Individual values of mV̇O_2_ (a) measured at rest and every minute during intermittent isometric contractions performed at 25% of MVC. (b) measured every minute during intermittent isometric contractions performed at 50% of MVC.

(*n*.)	mV̇O_2_ rest (μMol/s)	mV̇O_2_ – 1′ (μMol/s)	mV̇O_2_ – 2′ (μMol/s)	mV̇O_2_ – 3′ (μMol/s)	mV̇O_2_ – 4′ (μMol/s)	mV̇O_2_ – 5′ (μMol/s)	mV̇O_2_ – 6′ (μMol/s)
(a)							
S1	0.047	2.06	2.22	1.85	1.90	2.12	2.39
S2	0.021	0.87	1.79	2.77	2.38	1.09	1.81
S3	0.003	2.20	2.97	1.93	3.13	4.26	4.66
S4	0.043	1.28	1.29	1.49	1.59	1.76	1.65
S5	0.316	3.11	2.94	2.96	3.88	3.93	3.65
S6	0.002	2.06	1.90	2.31	1.99	2.03	2.08
S7	0.164	1.17	1.24	1.20	1.28	1.35	1.27
S8	0.089	2.80	2.80	2.69	2.65	2.86	2.28
S9	0.012	1.95	2.79	1.97	1.51	1.42	1.55
S10	0.048	1.41	1.08	1.30	1.35	2.02	2.33
Mean	0.07	1.89	2.10	2.05	2.16	2.28	2.37
SD	0.10	0.72	0.75	0.62	0.85	1.08	1.04

(*n*.)	mV̇O_2_ – 1′ (μMol/s)	mV̇O_2_ – 2′ (μMol/s)	mV̇O_2_ – 3′ (μMol/s)	mV̇O_2_ – 4′ μMol/s)	mV̇O_2_ – 5′ (μMol/s)	mV̇O_2_ – 6′ (μMol/s)
(b)						
S1	2.05	2.44	2.04	2.37	2.50	2.12
S2	2.82	1.83	1.61	3.40	2.03	2.17
S3	5.16	5.84	5.27	5.38	4.60	7.87
S4	1.51	2.07	1.86	1.92	2.31	1.96
S5	3.16	4.84	5.15	5.79	5.07	4.61
S6	1.86	1.31	1.79	2.23	1.15^a^	1.77^a^
S7	2.90	3.02	3.14	3.26	3.38	3.49
S8	3.16	2.69	1.62	3.37	4.73	4.34
S9	3.89	5.31	5.52	4.03	4.40	4.90
S10	2.69	3.42	3.12	2.65	2.72	3.50
Mean	2.92*	3.28*	3.11^#^	3.44*	3.29*	3.67*
SD	1.05	1.55	1.62	1.30	1.35	1.88
ES	1.14	0.97	0.87	1.16	0.83	0.86

*Note*: Mean values. Comparisons made by repeated‐measures two‐way ANOVA (mixed effects model) between the two % MVC *p < 0.05; ^#^0.05 < *p* < 0.1.

Abbreviations: ES, effect size Cohen's d; SD, standard deviation.

^a^ Excluded from comparison.

Repeated measures two‐way ANOVA revealed significant main effects of intensity (*F* (1, 18) = 6.05, *p* = 0.0242) and time (*F* (3.222, 56.70) = 2.753, *p* < 0.0472), no significant interaction between intensity and time (*F* (5, 88) = 0.2438, *p* = 0.9419). Post hoc pairwise comparisons showed that mV̇O_2_ was significantly higher at 50% MVC compared to 25% MVC at all time points except minute 3.

**FIGURE 2:**
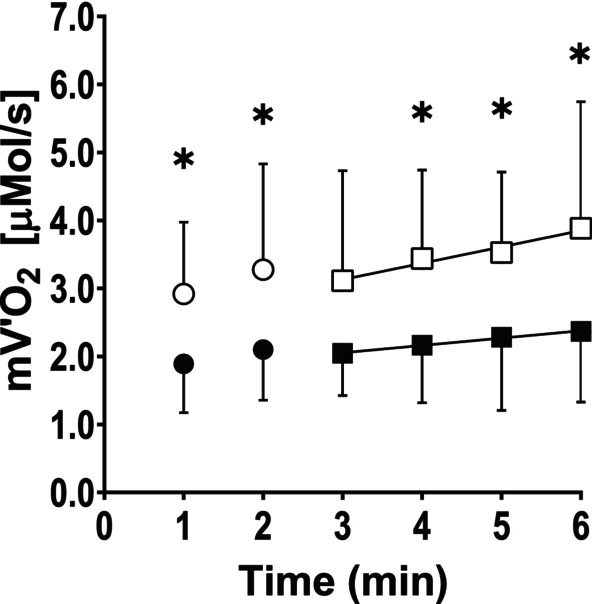
Average values of mV̇O_2_ as a function of time measured every minute from the onset of intermittent, submaximal isometric contractions carried out at 25% of MVC (black symbols) and 50% of MVC (empty symbols). Average mV̇O_2_ were linearly interpolated from the third to the sixth minute of exercise at the two intensities (squares symbols). *Significant difference between mV̇O_2_ at 50% and 25% MVC, *p* < 0.05. Please refer to the text for more details.

From the third minute of exercise onwards, mV̇O_2_ increased linearly at 25% of MVC (mV̇O_2_ = 1.73+ 0.108 × min; *r* ^2^ = 0.993; *p* < 0.0033; DF = 1, 2; *F* = 304) and at 50% of MVC (mV̇O_2_ = 2.41 + 0.240 × min; *r* ^2^ = 0.956; *p* < 0.0223; DF = 1, 2; *F* = 43.3). As expected, the slopes of the two linear regressions were significantly different (*p* < 0.0232; DF = 4; *F* = 12.8). These differences indicate that higher contraction intensities elicit a more pronounced slow component (Figure 2).

## 4 DISCUSSION

The results of the present investigation indicate that the progressive increase of mV̇O_2_ observed during a prolonged series of intermittent isometric contractions aligns with previous findings on HHb kinetics during sustained or intermittent exercise (Bringard & Perrey, 2004; Van Beekvelt et al., 2002) and can be assessed by measuring with NIRS the rate of rise of HHb concentration during brief complete arterial occlusions regularly interspersed in the series of contractions. While our study focused on evaluating mV̇O_2_ kinetics during intermittent isometric contractions using NIRS with arterial occlusions, similar approaches have been employed by several groups to assess mV̇O_2_ kinetics during recovery following exercise (Adami et al., 2017; Ryan et al., 2012; Zuccarelli et al., 2020). These studies demonstrated that NIRS is a reliable and reproducible method for quantifying muscle oxygen consumption dynamics in healthy individuals and clinical populations (e.g., COPD). In addition, the results suggest that the technique could discriminate between the rates of increase in mV̇O_2_ obtained by imposing different levels of isometric strength.

The linear increase in mV̇O_2_ observed from minute 3 onward reflects the progressive rise in muscle oxygen consumption likely associated with the slow component (V̇O_2sc_). This method aligns with previous studies on pulmonary and muscle‐level oxygen uptake kinetics (Grassi et al., 2015; Poole et al., 1991; Zoladz et al., 2008), where linear or exponential trends are commonly used to quantify slow‐component dynamics (Poole et al., 1991; Ryan et al., 2012).

### 4.1 Methodological considerations

Van Beekvelt et al. (2001) found a consistent difference between mV̇O_2_ measured with NIRS and that obtained by using Fick's law in combination with pulsossimetry, venous blood samples, and plethysmography during exercise (sustained isometric contraction at 10% of MVC), mV̇O_2_ measured with NIRS was consistently higher than the one assessed with Fick's law. Furthermore, the increase of mV̇O_2_, compared to the value prevailing at rest, was more significant with the first method than the second one. The results were attributed to the fact that the technique of Fick likely reflected the average mV̇O_2_ of the entire forearm, including the metabolic contribution of the muscles not directly recruited during the motor task. However, mV̇O_2_ measured with NIRS during arterial occlusions showed excellent repeatability (Van Beekvelt et al., 2001). They concluded that NIRS is a valuable and suitable tool for assessing local mV̇O_2_ of small muscular groups during specific motor tasks.

Furthermore, Hamaoka et al. (1996) showed strong correlations between NIRS‐derived measurements and phosphorus magnetic resonance spectroscopy (MRS), further confirming the validity of NIRS for investigating muscle oxygenation and metabolism in vivo. While our study did not directly compare mV̇O_2_ data obtained via NIRS with other techniques, these prior studies provide robust evidence supporting the methodological approach.

It has been underlined that NIRS cannot distinguish differences in HHb and reduced myoglobin (HMb) because of the identical absorption spectra of the two molecules. It is also controversial whether the NIRS signal originates from Hb (Seiyama et al., 1988; Wang et al., 1990) or Mb (Molé et al., 1999; Tran et al., 1999). Even though the quantification of the Mb contribution remains to be elucidated, ranging, according to some investigators, from about 10% (Seiyama et al., 1988) to about 70% during exercise (Davis & Barstow, 2013). However, this possible glitch would not substantially and negatively alter our data since we evaluated the volume of O_2_ consumed by the muscles independently from the source, either Hb or Mb. Yet, by expressing O_2_ turnover μmol/(L·s), we are interested in the amount of O_2_ taken up by the active tissue independent of its source, either HbO_2_ or MbO_2_ (Van Beekvelt et al., 2001).

Finally, we know that the NIRS light's penetration depth is limited to about 50% of the distance between the LED source and the detector. Therefore, a large ATT may be a substantial confounding factor in this measurement. The distance between optodes selected in the present investigation should have protected us from considerable bias.

V̇O_2peak_ was not measured because this study focused on localized muscle oxygen kinetics rather than systemic cardiorespiratory responses. Handgrip exercise involves a small muscle mass and does not elicit whole‐body oxygen consumption comparable to dynamic exercises. While our study focused on assessing mV̇O_2_ kinetics during intermittent isometric contractions using NIRS with arterial occlusions, similar approaches have been employed by several groups to evaluate mV̇O_2_ kinetics during recovery following exercise (Adami et al., 2017; Ryan et al., 2012; Zuccarelli et al., 2020).

Handgrip exercise involving a small muscle group was chosen to isolate local mV̇O_2_ dynamics and assess the slow component during intermittent isometric contractions. While this approach limits direct comparison with pulmonary V̇O_2_ kinetics due to the small muscle mass involved, it provides unique insights into local mechanisms underlying oxygen uptake kinetics. The methodology used here could be adapted for larger muscle groups or dynamic exercises in future studies to explore how local and systemic factors interact to produce the slow component.

### 4.2 Developments

The present results, obtained by using a noninvasive procedure that does not require expensive equipment or a highly trained operator, suggest that mV̇O_2_ can be assessed during repeated isometric muscular contractions.

In addition, applying techniques to small, superficial muscular groups might be successfully coupled with those capable of manipulating the number of motor units recruited in a motor task performed at a constant intensity to see how the muscular metabolic rate is affected.

### 4.3 Limitations

Our study specifically investigated mV̇O_2_ kinetics in a small muscle group (FDS) using NIRS during intermittent isometric contractions and does not account for systemic contributions or interactions between physiological systems, such as respiratory or cardiovascular components.

In addition, better quantification of Mb and Hb contributions to the NIRS signal remains to be elucidated in these exercise modalities. A longer exercise protocol would have exposed the volunteers to the risk of developing substantial fatigue, which would have prevented them from maintaining the imposed target load. However, it would have better characterized the time course of the muscular metabolic rate corresponding to the specific task.

## 5 CONCLUSIONS

In conclusion, we suggest that the slow component of mV̇O_2_, occurring during intermittent isometric contractions, can be assessed with NIRS during brief, complete arterial occlusions regularly interspersed in the series of contractions. In addition, the technique can discriminate the rates of increase in mV̇O_2_ corresponding to different percentages of MVC. Finally, because of its simplicity, the method, coupled with interventions designed to manipulate the number of motor units recruited in a motor task, may pave the way to a better understanding of the physiological and neuromuscular causes of V̇O_2sc_ in vivo.

## AUTHOR CONTRIBUTIONS

MB, CC, and ET were involved in conception of the study and design of the experiments and interpretation of the data. ET was involved in data collection. CC and ET were involved in analysis of the data and writing of the first draft. All authors were involved in revising the manuscript. All authors read and approved the final version of the manuscript.

## FUNDING INFORMATION

The study was supported by the funds for basic research allocated to the investigators by the Department of Neuroscience, Biomedicine and Movement Sciences, University of Verona, Verona.

## CONFLICT OF INTEREST STATEMENT

The authors declare no conflict of interest.

## ETHICS STATEMENT

The investigation has been conducted following the ethical standards of the Declaration of Helsinki. It has been approved by the author's institutional review board of the University of Verona (Italy) (*Comitato di Approvazione della Ricerca sulla Persona*—C.A.R.P.—n. 19.R2/2021 approval on 26th October 2021).

## Data Availability

The datasets generated during the current study are available from the corresponding author upon reasonable request.
